# Why clinical neuropsychology matters in schizophrenia care

**DOI:** 10.3389/fpsyg.2025.1713751

**Published:** 2025-12-05

**Authors:** Merete Glenne Øie, Fernando Barbosa, Sandra Lettner, Gus Baker, Cedric Duarte, Erik Hessen, Torill Ueland

**Affiliations:** 1 Department of Psychology, University of Oslo, Oslo, Norway; 2 Research Division, Innlandet Hospital, Brumunddal, Norway; 3 Laboratory of Neuropsychophysiology, Faculty of Psychology and Education Sciences, University of Porto, Porto, Portugal; 4 Austrian Society of Neuropsychology, Hohenzell, Austria; 5 Division of Neurosciences, University of Liverpool, Liverpool, United Kingdom; 6 Department of Psychology, Hôpitaux Robert Schuman, Luxembourg, Luxembourg; 7 Department of Neurology, Akershus University Hospital, Lørenskog, Norway; 8 Division of Mental Health and Addiction, Oslo University Hospital, Oslo, Norway

**Keywords:** schizophrenia, clinical neuropsychology, cognitive function, neuropsychological evaluation, subjective complaints, functional outcomes, neuropsychological rehabilitation

## Abstract

Cognitive impairments in schizophrenia are widespread, persistent throughout the illness course, and significantly impact quality of life and functional outcomes. Integrating neuropsychological evaluations into routine clinical care is essential for early identification of cognitive impairments. This approach facilitates the implementation of personalized treatment strategies, including tailored neuropsychological rehabilitation, to promote functional recovery. Although recommended by national and international guidelines, many individuals with schizophrenia do not receive neuropsychological assessment. This article emphasizes the integral role of clinical neuropsychology in schizophrenia care, focusing on six key areas: (1) cognitive domains to assess; (2) addressing subjective cognitive complaints; (3) the impact of symptoms, medication, and substance use on cognitive function; (4) test administration and evaluation procedures; (5) the utilization of neuropsychological results and feedback; and (6) the design and implementation of individualized neuropsychological rehabilitation.

## Introduction

Cognitive impairments are well documented features of schizophrenia, affecting up to 80% of individuals with the diagnosis (Vita et al., 2024). Impairments are documented across a wide range of cognitive domains, such as learning and memory, executive functioning, processing speed, and social cognition (Fett et al., 2011; Irani et al., 2012; McCutcheon et al., 2023). These impairments occur in premorbid and high-risk states, as well as during prodromal phases of the illness (Harvey et al., 2022; McCutcheon et al., 2023; Mohn-Haugen et al., 2022). Research supports both a generalized cognitive deficit and specific patterns of impairment that may vary across individuals and subtypes (McCutcheon et al., 2023). Cognitive impairments in schizophrenia are not merely a byproduct of positive psychotic symptoms or self-disorders; rather, they are more closely associated with negative symptoms and persist even when psychotic symptoms are in remission (Fett et al., 2022; Haug et al., 2023). Importantly, cognitive impairmets in individuals with schizophrenia are significantly linked to reduced real-world functioning (Kharawala et al., 2022).

Cognitive functioning in schizophrenia is heterogeneous, and accumulating evidence from data-driven clustering studies consistently identifies three distinct cognitive subtypes (Bechi et al., 2019; Gilbert et al., 2014; Rocca et al., 2016; Van Rheenen et al., 2017; Vaskinn et al., 2020; Weickert et al., 2000; Wells et al., 2015). The relatively intact subgroup, comprising roughly one-third of individuals, demonstrates performance close to normative levels. An intermediate subgroup, representing about 40–45% of individuals, shows moderate deficits across multiple cognitive domains. The severely impaired subgroup, accounting for approximately 20–30% of individuals, presents with global impairments often two to three standard deviations below the mean (Bechi et al., 2019; Gilbert et al., 2014; Rocca et al., 2016; Van Rheenen et al., 2017; Weickert et al., 2000; Wells et al., 2015). These profiles do not merely constitute cross-sectional differences; they also predict illness course, with the severely impaired group typically experiencing the poorest functional outcomes and the greatest symptom severity (Oomen et al., 2023; Vaskinn et al., 2020).

Cognitive impairments are prevalent across many mental disorders and are recognized as a transdiagnostic phenomenon (Abramovitch et al., 2021). Individuals with schizophrenia generally exhibit more severe widespread impairments compared to those with other mental disorders, including other psychotic disorders, although they do not have a unique neuropsychological profile (Abramovitch et al., 2021). A meta-analysis demonstrated a graded pattern of cognitive impairment across psychotic disorders, increasing from bipolar disorder to schizoaffective disorder and ultimately to schizophrenia (Lynham et al., 2022). While the affected cognitive domains are largely similar, the magnitude of impairment differs, highlighting primarily quantitative rather than qualitative differences, though heterogeneity within disorders suggests some qualitative distinctions. A recent systematic review and meta-analysis by Catalan et al. (2024) showed substantial variability across individuals with first-episode psychosis, including schizophrenia spectrum disorders. Variability was documented in core cognitive domains such as verbal learning, executive function, and processing speed, compared to healthy controls at baseline and follow-up. Importantly, cognitive heterogeneity encompasses both inter-individual differences and intra-individual fluctuations across tasks and time, particularly in processing speed, inhibitory control, verbal learning, and intellectual functioning (Haatveit et al., 2023). Increased variability, whether between or within individuals, is consistently linked to poorer functioning and more severe negative symptoms, underscoring the need for personalized cognitive assessment and intervention (Haatveit et al., 2023).

Cognitive impairments in schizophrenia are predominantly conceptualized within a neurodevelopmental framework, which posits that impairments originate early in development, frequently preceding the onset of clinical symptoms, and remain relatively stable across the course of illness as a consequence of atypical neurodevelopment (Kahn and Keefe, 2013). A longitudinal birth cohort study of cognitive functioning at ages 18 months, 4, 8, 15, and 20 years, including 4,322 participants, found that individuals who develop psychotic disorders exhibit a decline in verbal IQ during early childhood that then stabilizes. In contrast, full-scale and nonverbal IQ deficits continue to increase through adolescence and early adulthood, reflecting a growing lag behind typical developmental trajectories (Mollon et al., 2018). The findings suggest that psychotic disorders originate from dynamic neurodevelopmental processes affecting both verbal and nonverbal cognitive abilities throughout the first two decades of life, leading to increasing cognitive dysfunction. Importantly, these cognitive impairments are evident prior to the onset of psychosis, and while verbal IQ deficits tend to stabilize early, other cognitive domains, such as processing speed, working memory, and attention, may show slower growth or further decline extending into adolescence and early adulthood (Mollon et al., 2018). Furthermore, cross-sectional studies indicate that cognitive impairments primarily worsen during the transition from the prodromal phase to the first psychotic episode, with notable variability across different cognitive domains (Fett et al., 2022).

Longitudinal studies largely support that cognition is relatively stable across the course of illness, showing minimal average decline in cognitive performance across follow-up periods (Bergh et al., 2016; Flaaten et al., 2022). A comprehensive meta-analysis of 57 longitudinal studies in individuals with schizophrenia spectrum disorders further reported only modest improvements over time, most notably in processing speed (d = 0.32) and verbal memory (d = 0.21), with the largest gains in individuals with shorter illness duration (<10 years), suggesting relative stability beyond the early stages of illness (de Winter et al., 2024).

Nevertheless, certain subgroups deviate from this general pattern and show progressive decline in specific cognitive domains. This includes some older adults, who may experience accelerated cognitive aging, potentially related to neuroinflammation or the cumulative effects of chronic psychotic symptoms (Zipursky et al., 2013; Monji et al., 2013; Kharawala et al., 2022; McCleery and Nuechterlein, 2019). Additionally, individuals with early-onset schizophrenia may experience delayed cognitive maturation which can hinder development during critical periods (Øie et al., 2021). Thus, while global cognitive deficits typically appear early, further deterioration may occur in identifiable subgroups (Fett et al., 2020).

Despite the prevalence and persistence of cognitive impairment in schizophrenia, and recommendations in national and international guideline to conduct neuropsychological assessment, many individuals with schizophrenia do not receive such assessments (Bryce et al., 2024).

This article provides an overview of the critical role of neuropsychological evaluation in understanding and treating individuals with schizophrenia. It primarily focuses on neuropsychological assessment as a specialized clinical service for individuals with schizophrenia, targeting clinical neuropsychologists. As part of a multidisciplinary approach, neuropsychologists collaborate with psychiatrists and other clinicians to address the complex needs of individuals with schizophrenia (Gkintoni et al., 2024). Thus, this article also serves as a valuable resource for other healthcare professionals involved in the care and rehabilitation of these patients, particularly those who utilize neuropsychological findings or who conduct cognitive screening in their treatment of the patients.

Neuropsychological assessment is distinct from routine cognitive screening or research testing. It is a comprehensive evaluation conducted by trained neuropsychologists that integrates formal testing with behavioral observations, self-reports, and relevant medical and psychosocial context, including comorbidities and medication effects. This holistic method yields a nuanced understanding of cognitive strengths and weaknesses and guides personalized care decisions. Specifically, neuropsychological assessment examines relationships between brain function, behavior, and cognition, using a battery of tests assessing domains such as memory, attention, problem-solving, and language. These evaluations are essential for identifying cognitive deficits and resources, understanding their impact in psychiatric illnesses like schizophrenia, and informing treatment planning. By delineating this specialized evaluation process, this article also aims to bridge the gap between neuropsychological findings and their practical application among the diverse professionals involved in caring for individuals with schizophrenia.

The review in this article mainly emphasizes research on outpatient populations, where evaluation can inform functional recovery and community rehabilitation. While similar cognitive domains are examined in inpatient settings, logistical factors (e.g., symptom acuity, hospital milieu, and length of stay) and clinical priorities often differ (Kurebayashi and Otaki, 2018).

The goal is to enhance healthcare professionals’ understanding of the role of clinical neuropsychology and to promote its integration into standard care in schizophrenia. The specific topics covered include: (1) cognitive domains to assess; (2) addressing subjective cognitive complaints; (3) the impact of symptoms, medication, and substance use on cognitive function; (4) test administration and evaluation procedures; (5) the utilization of neuropsychological results and feedback; and (6) the design and implementation of individualized neuropsychological rehabilitation.

## Cognitive domains to assess

Cognitive impairments in individuals with schizophrenia are closely linked to functional impairment, emphasizing the need for neuropsychological assessment to guide interventions (Kharawala et al., 2022). On average, cognitive functioning in schizophrenia is approximately one standard deviation below the population mean, affecting various domains such as attention, processing speed, learning, working memory, long-term memory, executive function, motor skills, intellectual capacity, spatial reasoning, and social cognition, particularly theory of mind (Bora et al., 2017; Harvey et al., 2022; Vita et al., 2022). Impairments are most pronounced in memory and processing speed, while language, vocabulary, and spatial reasoning tend to be less severely affected (McCleery and Nuechterlein, 2019). Therefore, assessing these cognitive functions is crucial, as they directly impact the functioning and quality of life of individuals with schizophrenia.

Given that cognitive impairments can span multiple domains, comprehensive neuropsychological evaluations using standardized tests measuring a broad range of cognitive functions are essential. Identifying cognitive impairment is important, but understanding a patient’s cognitive strengths also informs therapeutic strategies and enhances quality of life (Allott et al., 2020a). Furthermore, while many individuals with schizophrenia may perform below average on assessments, some can achieve normal-range scores yet still face significant challenges in areas like attention and executive functioning. These difficulties can hinder effective use of cognitive resources in daily activities and social interactions (Vaskinn et al., 2020). Therefore, employing multiple tests is critical to capture the full spectrum of cognitive strengths and weaknesses for effective rehabilitation.

To evaluate cognitive functions, standard neuropsychological tests can be employed, utilizing both collections of individual tests and established test batteries. An advantage of standardized test batteries is that they often cover many cognitive dimensions and share common norms. A test battery frequently used in schizophrenia research is the Measurement and Treatment Research to Improve Cognition in Schizophrenia Consensus Cognitive Battery (MCCB), commonly known as MATRICS (Nuechterlein et al., 2008). However, it is important to note that the MATRICS battery is not typically used in clinical practice. This battery assesses key cognitive domains through ten distinct tests, selected based on extensive research into their relevance for understanding cognitive impairments associated with schizophrenia. Extensive research underscores the close connection between these cognitive domains and functional outcomes in individuals with schizophrenia (Green et al., 2004), reinforcing their significance for quality of life, employment, social integration, and independent living (August et al., 2012). The MCCB evaluates seven cognitive domains: processing speed (rapid information processing and response), attention/vigilance (the ability to maintain focus over time), working memory (the temporary holding and manipulation of information), verbal learning (acquisition and recall of verbally presented information), visual learning (acquisition and recall of visual presented information), reasoning and problem-solving (logical thinking and problem-solving abilities), and social cognition (understanding social interactions and the intentions of others) (Nuechterlein et al., 2008).

While the MATRICS provides a structured and research based approach to understanding cognitive impairments, the battery does have some limitations. Several domains are represented by a single measure, which may compromise reliability, and the verbal and visual learning subtests lack delayed recall components, restricting the evaluation of memory processes. Additionally, costs and training requirements can limit its practicality in everyday clinical environments (Nuechterlein et al., 2008; August et al., 2012; Nuechterlein et al., 2025). However, clinicians often adapt MATRICS-guided evaluations using locally available instruments to ensure comprehensive, flexible, and contextually relevant assessments.

Clinicians should consider the availability and applicability of comprehensive test batteries for clinical use (Nuechterlein et al., 2025). The Cambridge Neuropsychological Test Automated Battery (CANTAB) has been utilized in cognitive studies of schizophrenia and is recommended as an alternative to the MCCB. This computerized battery, administered on touch-sensitive screens, provides a non-language-based approach that effectively identifies cognitive impairments addressing working memory, decision-making, attention, executive functions and visual memory in schizophrenia (Fray et al., 1996; Levaux et al., 2007). CANTAB’s is good choice in clinical settings, often producing results comparable to those from traditional neuropsychological tests (Levaux et al., 2007).

While larger test batteries are valuable for measuring a wide range of cognitive functions, shorter test batteries are particularly important for some patients with schizophrenia, as negative symptoms may lead to reduced motivation, impaired engagement, and lower endurance for lengthy cognitive testing (Strauss et al., 2015). Therefore, test durations should be tailored to each patient, with sessions divided as needed. Alternatively, one might start with a brief cognitive screening and refer patients with identified cognitive impairments to a comprehensive neuropsychological evaluation.

Examples of shorter test batteries (25–30 min) are the Brief Assessment of Cognition in Schizophrenia (BACS; Keefe et al., 2004) and the Repeatable Battery for the Assessment of Neuropsychological Status (RBANS; Randolph et al., 1998). They are easy to administer and score, cost-effective, and suitable for use across different cultural contexts. Both batteries have demonstrated strong reliability and validity in schizophrenia populations, with the BACS specifically developed for cognitive assessment in schizophrenia. Its reliability, validity, and comparability of original forms have been established empirically (Keefe et al., 2004). The BACS composite score also shows strong functional validity, correlating with measures of everyday functioning such as independent living and performance-based skills (Keefe et al., 2006).

Originally designed as a brief neuropsychological screening tool for dementia (Randolph et al., 1998), the RBANS has also proven effective and valid for detecting cognitive impairment in schizophrenia, with good reliability and comparability to more extensive cognitive batteries (Gold et al., 1999; Dickerson et al., 2004; Raudeberg et al., 2023). Additionally, shorter assessments can be administered by a wider range of clinical staff, reducing the need for highly specialized training (see Nuechterlein et al., 2025 for a discussion of test batteries used for neuropsychological assessment in schizophrenia).

Finally, performance validity testing should be routinely included in neuropsychological assessments of individuals with schizophrenia, as insufficient effort or invalid test results can lead to inaccurate cognitive evaluations and affect treatment planning (Morra et al., 2015). A meta-analysis of 19 studies involving over 2,200 individuals with psychotic disorders revealed a pooled effort failure rate of 18%, with substantial variability across studies (ranging from 0 to 72%) (Ruiz et al., 2020). The results indicated that lower IQ and fewer years of education significantly increased the risk of failure, suggesting that cognitive capacity and demographic factors, rather than malingering, are primary contributors to reduced effort. These findings underscore the need for careful interpretation of performance validity measures in individuals with schizophrenia, taking into account the broader context of global cognitive impairment and clinical presentation. When employing effort tests for this population, it is recommended to use multiple measures and adopt a comprehensive decision-making approach that integrates various sources of information, such as clinical interviews, behavioral observations, referral data, and effort test performance (Ruiz et al., 2020) (see Box 1).

Box 1 • Thorough neuropsychological evaluations of functions such as attention, processing speed, motor skills, learning/memory, executive function, intellectual capacity, and social cognition are essential for accurately identifying cognitive strengths and difficulties in individuals with schizophrenia.

• Given the variability in cognitive functioning, comprehensive evaluations across multiple domains are highly recommended.

• Screening tools can be useful, but detailed evaluations are necessary for a broader understanding and for developing tailored interventions.

## Assessment of subjective cognitive complaints

Individuals with schizophrenia often report subjective cognitive complaints, which are also noted by their family members (Bryce et al., 2024, 2025; Wood et al., 2015). These complaints can have a negative impact on self-esteem and quality of life and might be overlooked if assessments rely on objective measures alone (Shin et al., 2016; Wood et al., 2015). Therefore, incorporating subjective complaints into evaluations is important. Research shows weak correlations between subjective and objective cognitive functioning in non-clinical individuals, suggesting that standardized objective tests may not fully capture individual challenges (Grimstad et al., 2025; Haddad et al., 2021; Rothlind et al., 2017). Subjective reports may offer a more ecologically valid assessment by better reflecting the complexity of everyday situations than structured objective tests.

However, accurately capturing subjective complaints can be challenging. High levels of depression are linked to underestimation of cognitive abilities in patients with schizophrenia (Baliga et al., 2020). Furthermore, accurate self-reporting requires a certain level of cognitive functioning, such as the ability to recall daily memory problems (Nuechterlein et al., 2025). Research indicates that individuals with schizophrenia often have impaired insight into their illness, including their cognitive functioning (Haddad et al., 2021; Nuechterlein et al., 2025). This lack of insight, coupled with higher levels of disorganized symptoms, may contribute to difficulties in self-assessing cognitive abilities (McCutcheon et al., 2023). Thus, when insight is reduced, supplementing self-reports with information from others familiar with the individual’s functioning, such as family, caregivers, case managers, treatment providers, or friends, can be beneficial.

In addition, Haugen et al. (2021) found that patients with schizophrenia with low self-efficacy tended to report greater cognitive difficulties than their test performance suggested, whereas those with more disorganized symptoms often overestimated their abilities. Clinicians can use this information to tailor interventions, such as fostering self-efficacy in individuals who underestimate their capacities or enhancing cognitive insight in those who overestimate their skills (Haugen et al., 2021).

Despite potential biases, subjective assessments remain valuable for understanding how patients view their abilities and challenges. Notably, some studies show that many patients can accurately recognize their impairments (Haddad et al., 2021; Raffard et al., 2020). For patients with good insight into their cognitive difficulties, assessing subjective experiences can provide critical information about how these challenges affect their daily functioning and self-efficacy. Such insights enable clinicians to prioritize interventions that align with the patient’s perceived difficulties, enhance engagement in cognitive remediation, and support collaborative goal setting. For instance, when patients can clearly articulate situations in which they struggle with concentration or memory, clinicians can tailor cognitive strategies to these specific contexts, promoting both adherence and functional improvement. Integrating subjective experiences with objective assessments provides a more comprehensive understanding of a patient’s cognitive profile, enabling personalized and meaningful interventions that strengthen the therapeutic relationship, enhance motivation, and improve functional outcomes (Allott et al., 2020a).

Several scales have been developed to measure subjective cognitive complaints in individuals with schizophrenia. However, several of them also assess other symptoms. Two, which specifically focus only on cognition, are the Subjective Scale to Investigate Cognition in Schizophrenia (SSTICS; Stip et al., 2003) and the Self-Assessment Scale of Cognitive Complaints in Schizophrenia (SASCCS; Johnson et al., 2009).

The SSTICS includes 21 items rated from 0 (never) to 4 (very often) on a Likert scale, each assessing perceived difficulties in attention, memory, executive functions, language, and praxia; total scores are calculated by summing ratings across items, with higher scores indicating greater subjective cognitive impairment (Stip et al., 2003). The SASCCS also consists of 21 items focused on similar domains, placing emphasis on attention and executive functioning (Johnson et al., 2009). Both scales are brief self-reports completed in less than 10 min. More recently, a brief version of the SSTICS (SSTICS-Brief; Cella et al., 2020) was developed to further simplify assessment while maintaining robust validity.

These instruments are reliable for measuring patients’ perceptions of their own cognitive impairment; although they should not be a substitute for objective cognitive assessment. Their primary value lies in permitting patients to express their perceived well-being and satisfaction with quality of life, offering clinicians insight into both psychological and functional aspects of schizophrenia (Johnson et al., 2009). Subjective cognitive complaints assessed through these scales may influence clinical decision-making, guide interventions, and help monitor changes in quality of life over time.

Additionally, interview-based assessments such as the Schizophrenia Cognitive Rating Scale (SCoRS) and the Cognitive Assessment Interview (CAI) offer valuable insights by integrating both patient and informant reports (Harvey et al., 2019; Ventura et al., 2013). These tools are advantageous due to their relatively short administration times, validation across various languages, and strong correlations with functional and cognitive performance measures (see Nuechterlein et al., 2025, for a summary of the main interview-based neuropsychological assessments currently in use) (see Box 2).

Box 2 • Objective measures assess cognitive performance, while subjective complaints highlight real-life functioning.

• Incorporating assessment of subjective cognitive complaints into the evaluation provides a more holistic understanding of the individual’s cognitive function.

• Co-morbid symptoms, memory problems, and lack of insight can bias subjective assessments; therefore, input from caregivers and other relevant informants is invaluable.

## Impact of symptoms, medication, substance use and other potential confounders

Several factors can potentially impair cognitive functioning, regardless of whether an individual has a mental disorder or not. Below, we outline possible mediators and moderators of cognitive test performance that are particularly important to consider during the neuropsychological evaluation of individuals with schizophrenia (Moritz et al., 2021).

### Symptoms

Research shows that positive symptoms, such as hallucinations and delusions, have little effect on cognitive functioning (McCutcheon et al., 2023; Palmer et al., 2009). In contrast, negative symptoms, such as blunted affect, social withdrawal, and apathy are significantly associated with greater cognitive impairment and influence the relationship between cognitive performance and functional outcomes (Bora et al., 2023; Kharawala et al., 2022). Thus, an evaluation of negative symptoms should be included for accurate interpretation of cognitive assessment results.

Standardized instruments for assessing negative symptoms include the Positive and Negative Syndrome Scale (PANSS) (Kay et al., 1987), the Scale for the Assessment of Negative Symptoms (SANS) (Andreasen, 1981), the Brief Negative Symptom Scale (BNSS) (Kirkpatrick et al., 2011), and the Clinical Assessment Interview for Negative Symptoms (CAINS) (Kring et al., 2013). It is also important to note that significant cognitive difficulties can persist even when psychotic symptoms are minimal (Reichenberg, 2010).

Many individuals with schizophrenia also have comorbid conditions such as depression (Etchecopar-Etchart et al., 2021), which can worsen cognitive impairment (Abramovitch et al., 2021). Hence, it is essential to also consider these comorbidities when interpreting cognitive test results, as they can confound the assessment of cognitive functioning and impact functional outcomes.

### Medication use

Antipsychotic medications effectively reduce psychotic symptoms that interfere with attention, yet their direct impact on cognition is modest. A recent meta-analysis of 28 double-blind randomized controlled trials (n = 1,932) found that haloperidol, a first-generation antipsychotic, was associated with slightly poorer overall cognition and performance in several domains, processing speed, attention, motor function, memory and verbal learning, and executive function, compared to second-generation antipsychotics (SGAs), although differences were small (Baldez et al., 2025). No statistically significant differences were observed for working memory, visual learning, social cognition, or visuoconstruction. Thus, while SGAs may offer small cognitive advantages over haloperidol in certain domains, the clinical relevance of these differences remains uncertain (Baldez et al., 2025). A network meta-analysis of 68 studies (n = 9,525) found no antipsychotic to be superior to placebo in improving cognitive performance, with first-generation agents and clozapine ranking among the lowest. Cognitive assessments across studies were frequently inconsistent and incomplete (Feber et al., 2025).

Medications with anticholinergic or antihistaminergic properties can induce sedation and daytime drowsiness, which may adversely affect cognitive functioning (Harvey et al., 2022; Vita et al., 2024). Further, high doses and polypharmacy, especially those resulting in elevated D2 receptor occupancy, can also impact cognition. These medications often induce significant metabolic side effects, including obesity, dyslipidemia, and type 2 diabetes, which are associated with an increased risk of cognitive impairment, rather than being direct causes themselves (MacKenzie et al., 2018). Additionally, evidence suggests that certain structural brain changes linked to antipsychotic use may further complicate the relationship with cognitive function (Fusar-Poli et al., 2013; Voineskos et al., 2020). Maintaining antipsychotic doses within a normal to low range while avoiding polypharmacy appears to yield a slightly favorable effect on cognitive performance (Haddad and Correll, 2023). It is important to note that patients also report variable impacts of medication on cognitive functioning. Thus it is important to also consider conducting a medication review if adverse effects are observed or experienced (Allott et al., 2025).

Finally, a meta-analysis of 23 studies demonstrated similar cognitive impairments in drug-naive patients as those observed in patients treated with antipsychotics, indicating that medications are not the sole contributors to cognitive impairments (Fatouros-Bergman et al., 2014).

### Substance use

Substance use disorders are highly prevalent among individuals with schizophrenia, with lifetime rates estimated at 20–65% (Ward et al., 2023) and approaching 80% when tobacco use is included (Manseau and Bogenschutz, 2016). Both schizophrenia and substance use are independently associated with impairments in cognitive functioning, and the co-occurrence of these conditions can intensify such impairments. Commonly used substances, including nicotine, caffeine, alcohol, cannabis, and cocaine, may exacerbate cognitive impairment by disrupting neurotransmitter systems, particularly dopaminergic, glutamatergic, and GABAergic pathways (Bruijnen et al., 2019).

Based on the comprehensive review by McCutcheon et al., 2023, acute cannabis use is generally associated with cognitive impairment, and regular cannabis users tend to perform worse on cognitive tests. However, the effects of cannabis use on cognition in schizophrenia are complex and inconsistent. For example, Rachid et al. (2023) found that patients with schizophrenia who did not use cannabis performed better in psychomotor function, attention, and verbal memory, while cannabis users showed better performance in working memory, visual memory, and emotional recognition, with no differences observed in executive function. Possible explanations for better performance among some cannabis-using patients include less severe baseline cognitive impairment, greater motivation and organisational skills, and a lower genetic liability for schizophrenia (See McCutcheon et al., 2023). Cannabidiol (CBD) may also exert neuroprotective effects, although most illicit cannabis today contains high levels of delta-9-tetrahydrocannabinol (THC) and little CBD. THC is known to impair attention, memory, and executive function, and may exacerbate psychotic symptoms (See McCutcheon et al., 2023). Together, these findings suggest that cannabis may have differential effects on distinct cognitive domains in schizophrenia.

Similar variability is observed with other substances; while many studies in dual-diagnosis populations show impairments in executive function, others report comparable or even superior processing speed relative to non-using patients with schizophrenia (Thoma and Daum, 2013). Such differences may reflect the type of substance used, its pattern and severity, and individual illness characteristics. Alcohol abuse, in particular, is consistently linked to more severe deficits (Thoma and Daum, 2013).

When assessing cognition in individuals with schizophrenia and comorbid substance use disorders, it is essential to consider characteristics of the substance use itself, such as severity, duration, frequency of relapse, and periods of abstinence, as well as psychosocial functioning (Adan et al., 2017). Poly-substance abuse is particularly concerning, as it has been associated with persistent cognitive deficits and minimal recovery even after 1 year (Medina et al., 2004). These factors highlight the need for comprehensive evaluation to understand the complex and multifactorial relationship between substance use and cognitive impairment. Ideally, neuropsychological testing should be conducted after a sustained period of abstinence. However, when abstinence is not achievable, assessments can still inform treatment strategies as long as the patient is not acutely intoxicated and is able to understand instructions.

### Other potenstial confounders

There are also other factors that can affect cognitive function in schizophrenia. Metabolic and cardiovascular conditions, such as metabolic syndrome, hypertension, and diabetes, are common in individuals with schizophrenia and are associated with impaired cognitive performance (Boussaid et al., 2024; Hagi et al., 2021; Vancampfort et al., 2015). Sleep disturbances, frequently reported among individuals with schizophrenia, are also linked to cognitive impairments (Ferrarelli, 2021; Laskemoen et al., 2020). Anxiety and depressive symptoms are prevalent in this patient group, affecting mood and motivation, potentially leading to reduced cognitive function (Buckley et al., 2009; Moritz et al., 2021).

Vita et al. (2024) differentiate between “primary cognitive impairment,” resulting from neurobiological changes inherent to schizophrenia, and “secondary cognitive impairment,” arising from external factors such as symptoms and lifestyle. While secondary factors, such as symptoms, medication side effects, or lifestyle factors, can lead to cognitive improvement, primary impairments require targeted therapeutic approaches that directly treat the core cognitive deficits of schizophrenia. By providing thorough evaluations considering both types of impairments, neuropsychologists can guide more effective treatment planning for individuals with schizophrenia (see Box 3).

Box 3 • Negative symptoms are significantly associated with cognitive impairments, whereas positive symptoms have a weaker relationship.

• Antipsychotic medications do not lead to clinically significant improvements in cognitive functioning but may impair functioning due to anticholinergic properties or side effects.

• Assessing the potential impact of substance use, symptoms, medication side effects, sleep and circadian abnormalities, and comorbid conditions, is important.

## Evaluation procedures and test administration

While cognitive screening can be carried out by experienced psychologists who are not specialists, it is essential that these assessments are supervised by clinical neuropsychologists to maintain precision and reliability. Importantly, clinical neuropsychological assessments should not be reduced to mere testing. It is a multifaceted process that encompasses self-reporting, behavioral observations, external exploration, and formal testing, while also accounting for comorbidities, medication effects, brain lesions, and other influencing factors. This paradigm underscores the complexity of a neuropsychological evaluation, which extends beyond simple test scores to provide a holistic understanding of the patient.

Neuropsychological testing should be avoided during the acute psychotic phase, characterized by symptoms such as intense hallucinations, delusions, and disorganized speech or behavior. Conducting assessments during this period can obscure the individual’s true neuropsychological status and hinder the accurate identification of their cognitive strengths and weaknesses. Additionally, patients must be able to concentrate adequately to understand task instructions and perform at their best, which is often difficult when experiencing acute psychosis.

Cognitive functioning in schizophrenia can fluctuate over time due to factors such as symptom exacerbations, medical treatment effects, and psychosocial interventions. Longitudinal assessments are valuable for tracking these changes and adjusting treatment plans accordingly. Continuous collaboration with other healthcare professionals in the treatment team is essential to determine the optimal timing for testing each patient (see Box 4).

Box 4 • Schedule neuropsychological testing when the patient is clinically stable, and ensure the testing environment is comfortable and supportive to help minimize anxiety.

• Consider shorter assessments with frequent breaks to accommodate fatigue and motivation challenges.

## Utilization of neuropsychological evaluation and feedback in schizophrenia

Providing structured and accessible feedback from neuropsychological evaluations is essential for all patients undergoing assessment. However, it plays a particularly critical role in individuals with schizophrenia, where cognitive deficits significantly affect daily functioning. To maximize understanding and facilitate meaningful discussions about cognitive functioning, practitioners should avoid jargon and focus on practical implications, linking cognitive performance to daily life challenges and specific goals (Bryce et al., 2025).

As many people with schizophrenia rely heavily on family support, clear communication with both patients and caregivers helps align expectations and improve understanding of illness-related cognitive challenges. Additionally, incorporating psychoeducation into the feedback process can enhance caregivers’ ability to support patients, providing them with strategies to address cognitive difficulties and fostering better engagement in treatment (Bryce et al., 2025; Glecia and Li, 2024).

Neuropsychological reports for this population should clearly outline both cognitive strengths and limitations to guide personalized, targeted interventions. Feedback should be tailored to the individual’s level of insight and incorporate both objective test results and the patient’s subjective experience, as both substantially influence functional outcomes in schizophrenia (Harvey, 2012). When providing feedback, it is crucial to present information in a clear and empathetic manner, ensuring that patients understand their cognitive strengths and areas for improvement (Bryce et al., 2025). Additionally, utilizing visual aids and written summaries can enhance comprehension and retention of information, which is particularly beneficial for individuals with cognitive impairments (Gruters et al., 2022).

For patients with schizophrenia, recommendations should explicitly address how cognitive impairments affect everyday responsibilities, such as medication adherence, financial management, or caregiving for dependents, and propose practical, specific solutions. These may include environmental adaptations, compensatory strategies (e.g., reminders, structured routines), psychosocial interventions, and cognitive rehabilitation targeted to domains commonly affected in schizophrenia, such as attention, memory, and executive function (Zoupa et al., 2023).

It is also important to identify and address external, modifiable factors, such as poor sleep, physical inactivity, medication side effects, stress, and social isolation, that can further undermine cognition in this population (Moritz et al., 2021). Support from welfare agencies, occupational therapists, and assistive technologies can further enhance functioning.

Finally, neuropsychological evaluations in schizophrenia can help clinicians provide realistic yet optimistic guidance regarding a patient’s potential for employment and education. They can also improve the effectiveness of therapeutic approaches, such as talk therapy and psychoeducation, by adapting them to the patient’s cognitive profile. For example, therapy sessions that are shorter, more structured, and include multimodal presentation of information can yield better outcomes for individuals with schizophrenia who experience memory or attention deficits (Buckley et al., 2009) (see Box 5).

Box 5 • Ensure that feedback is structured and straightforward, emphasizing both cognitive strengths and weaknesses.

• Provide practical suggestions, such as making environmental adjustments, utilizing compensatory strategies, and addressing modifiable factors like sleep and stress.

• Involve caregivers to help them understand the patient’s cognitive strengths and weaknesses, enabling them to adjust their expectations accordingly.

## Neuropsychological rehabilitation

Neuropsychological rehabilitation is an integrated, goal-oriented strategy designed to enhance cognitive, emotional, psychosocial, and behavioral functions through collaboration among patients, their families, and healthcare professionals. In schizophrenia, this approach is important for improving real-world functional outcomes and supporting patients in a personalized and holistic manner (Zoupa et al., 2023). The implementation of targeted interventions to improve cognitive function should involve a multidisciplinary team comprising clinical neuropsychologists, psychologists, psychiatrists, nurses, occupational therapists, and social workers. Caregiver involvement is also essential, as they play an active role in supporting treatment adherence (Bryce et al., 2025).

Cognitive remediation is an evidence-based treatment for cognitive impairments in schizophrenia. It is defined as “a behavioral training intervention targeting cognitive deficits, using scientific principles of learning, with the ultimate goal of improving functional outcomes” (Cognitive Remediation Experts Working group (CREW), 2010). Although cognitive remediation programs vary with regard to techniques employed, they share four common principles. These include having a trained therapist deliver the program, repeated practice of cognitive exercises, development of problem-solving strategies, and incorporating procedures that facilitate the transfer of skills to real-world functioning (Bowie et al., 2020).

An example of a targeted cognitive remediation program is the Thinking Skills for Work program (TSW) (McGurk and Mueser, 2021). The TSW features computerized cognitive exercises and teaching cognitive self-management strategies, delivered by trained cognitive specialists in conjunction with supported employment services. The TSW program has shown improved cognitive functioning and employment outcomes in multiple studies (McGurk et al., 2007; McGurk et al., 2015). Other targeted programs include CIRCuiTS (Reeder et al., 2016), the Neuropsychological Educational Approach to Cognitive Remediation (NEAR) (Medalia and Freilich, 2008), and Cognitive Symptom Management and Rehabilitation Training (CogSMART) (Twamley et al., 2012). More information about these and other relevant programs can be found at.^1^ Numerous studies confirm that cognitive remediation has positive effects on cognitive impairments and functional outcomes in schizophrenia, supporting its integration into clinical practice and guidelines (Harvey et al., 2022; Kambeitz-Ilankovic et al., 2019; Lejeune et al., 2021; Vita et al., 2021; Wykes et al., 2011). Notable cognitive effects include improvements in global cognition, verbal learning, and working memory, with more modest benefits for attention and processing speed (Fitapelli and Lindenmayer, 2022). Evidence suggests that approaches emphasizing strategy development and metacognitive training are particularly effective in improving cognitive outcomes (Fitapelli and Lindenmayer, 2022; Wykes et al., 2011; Haugen et al., 2022).

The impact of cognitive remediation on functioning has been shown to be particularly effective when integrated with other psychosocial rehabilitation approaches such as supported employment (McGurk et al., 2007; McGurk et al., 2015). The feasibility of cognitive remediation, even during the acute phases of schizophrenia, highlights its broad applicability (Nemoto et al., 2021). Research by Harvey et al. (2022) indicates that effect sizes for global cognition and functioning in cognitive remediation for schizophrenia range from small to moderate, with outcomes influenced by factors such as metabolic syndrome, lower IQ, older age, and genetic polymorphisms. This variation underscores the need to optimize treatment strategies by incorporating complementary interventions. Integrating physical exercise into rehabilitation programs may enhance neuropsychological performance and complement psychosocial interventions (Dai et al., 2022; Firth et al., 2017). Moreover, Vita et al. (2024) suggest that an integrated approach combining cognitive remediation, physical exercise, neurostimulation, and antipsychotic medication, tailored to individual needs, may offer the best results.

To further enhance these approaches, it is important to provide support that helps individuals compensate for cognitive impairments. This involves employing internal techniques like memory strategies and external tools such as planners, apps, or assistive devices. A recent review and meta-analysis found that such compensatory strategies significantly and durably improve functioning in individuals with psychotic disorders (Allott et al., 2020b). Equally important are environmental adaptations at home, school, and work to maximize cognitive resources, thereby supporting enhanced functional outcomes.

Finally, efficient neuropsychological rehabilitation in schizophrenia must also address broader aspects of psychological and physical health, social life, and overall quality of life. Integrating psychosocial treatments can enhance adherence to treatment, reduce rehospitalization rates, and improve social cognition, indirectly benefiting cognitive outcomes (Rampino et al., 2021; see Box 6).

Box 6 • Emphasize the role of environmental adaptation and compensation strategies, to enhance daily functioning.

• Collaborate within a multidisciplinary team to enhance the efficacy of neuropsychological rehabilitation.

• If available, consider referring to a cognitive remediation program.

### Case example

This case illustrates the benefits of a neuropsychological evaluation by a clinical neuropsychologist and subsequent neuropsychological rehabilitation for individuals with schizophrenia. It highlights the critical role of multidisciplinary support in improving daily functioning and overall quality of life.

Ann, a 34-year-old diagnosed with schizophrenia and undergoing antipsychotic treatment, has been hospitalized four times. She attends day treatment twice a week and lives with her supportive parents, who notice her difficulties with daily activities like getting up in the morning, missing appointments, and feeling isolated. A recent neuropsychological evaluation revealed that Ann has a normal IQ but struggles with memory and concentration.

Her treatment involved a multidisciplinary team, including a clinical neuropsychologist, psychiatrist, occupational therapist, and physiotherapist. The psychiatrist managed her medication carefully to avoid neuropsychological side effects. Ann participated in exercise groups run by a local physiotherapist, which helped improve her sleep problems. She joined a goal management training group with other patients, where she received support in achieving everyday goals such as attending social appointments. Ann was encouraged to use tools like diaries for planning, mobile reminders for appointments, and post-it notes for her morning routine, allowing her to handle tasks independently. She also learned techniques like verbalizing and repeating information to aid memory and enhance social interactions, improving focus, memory, and task completion.

Initially, Ann feared that her psychotic episodes and past antipsychotic treatments had damaged her memory and concentration. However, the neuropsychological evaluation provided verbal and written reassurances that her difficulties would not likely worsen and that strategies could help compensate. This boosted her self-confidence and reassured her about her normal intellectual abilities, reducing the shame of memory lapses during social interactions. As her daily functioning improved, reintegration into the work environment became a key goal in Ann’s rehabilitation. Her treatment team helped Ann seek jobs that matched her neuropsychological profile. This approach not only supports Ann’s transition back to work but also helps prevent potential disappointments, making her reintegration a more positive experience and an important step toward greater participation in life.

## Conclusion

Clinial neuropsychology plays a crucial role in managing and planning treatment for individuals with schizophrenia. By identifying cognitive strengths and weaknesses, neuropsychological evaluations can guide the development of personalized intervention strategies, which can enhance daily functioning and improve quality of life. In the long term, the work of clinical neuropsychologists is cost effective in schizophrenia care, benefiting both individuals and society by improving functional outcomes and reducing healthcare costs.

The unique expertise of clinical neuropsychologists lies in their ability to evaluate cognitive functioning using reliable, objective, and valid psychometric tests. In the interpretation of test-results, neuropsychologists are well trained to consider factors such as functional neuroanatomy, comorbid diseases, and disruptive influences like medication and psychotic phases. Providing clear and meaningful feedback on the results of a neuropsychological evaluation is a vital component of clinical neuropsychology, as it informs other mental health professionals, empowers patients, and guides treatment planning.

We advocate for the routine integration of neuropsychological evaluations into the assessment process and treatment plans for patients with schizophrenia. By doing so, clinical neuropsychologists can not only enhance patient care but also serve as a valuable resource for other healthcare professionals involved in the treatment and rehabilitation of individuals with schizophrenia. This comprehensive and economically sustainable approach leverages the full potential of clinical neuropsychologists to improve patient outcomes.

## Footnotes

1
https://www.cognitiveremediation.info/

## Group members of EFPA’s Standing Committee on Clinical Neuropsychology

Gus Baker, Fernando Barbosa, Marios Constantinou, Cédric Duarte, Kristina Hedman, Erik Hessen, Laura Hokkanen, Maria K. Jonsdottir, Erich Kasten, Mary Helen Kosmidis, Sandra Lettner, Sara Mondini, Niall Pender, Amélie Ponchel, Aistė Pranckevičienė.
